# EpiVar Browser: advanced exploration of epigenomics data under controlled access

**DOI:** 10.1093/bioinformatics/btae136

**Published:** 2024-03-06

**Authors:** David R Lougheed, Hanshi Liu, Katherine A Aracena, Romain Grégoire, Alain Pacis, Tomi Pastinen, Luis B Barreiro, Yann Joly, David Bujold, Guillaume Bourque

**Affiliations:** Canadian Centre for Computational Genomics, McGill University, Montreal, QC H3A 0G1, Canada; Department of Human Genetics, McGill University, Montreal, QC H3A 0G1, Canada; Victor Phillip Dahdaleh Institute of Genomic Medicine at McGill University, Montreal, QC H3A 0G1, Canada; Department of Human Genetics, McGill University, Montreal, QC H3A 0G1, Canada; Victor Phillip Dahdaleh Institute of Genomic Medicine at McGill University, Montreal, QC H3A 0G1, Canada; Centre of Genomics and Policy, McGill University, Montreal, QC H3A 0G1, Canada; Department of Human Genetics, University of Chicago, Chicago, IL 60637, United States; Canadian Centre for Computational Genomics, McGill University, Montreal, QC H3A 0G1, Canada; Canadian Centre for Computational Genomics, McGill University, Montreal, QC H3A 0G1, Canada; Victor Phillip Dahdaleh Institute of Genomic Medicine at McGill University, Montreal, QC H3A 0G1, Canada; Department of Human Genetics, McGill University, Montreal, QC H3A 0G1, Canada; Victor Phillip Dahdaleh Institute of Genomic Medicine at McGill University, Montreal, QC H3A 0G1, Canada; Genomic Medicine Center, Children's Mercy, Kansas City, MO 64108, United States; Department of Human Genetics, University of Chicago, Chicago, IL 60637, United States; Section of Genetic Medicine, Department of Medicine, University of Chicago, Chicago, IL 60637, United States; Committee on Immunology, University of Chicago, Chicago, IL 60637, United States; Department of Human Genetics, McGill University, Montreal, QC H3A 0G1, Canada; Victor Phillip Dahdaleh Institute of Genomic Medicine at McGill University, Montreal, QC H3A 0G1, Canada; Centre of Genomics and Policy, McGill University, Montreal, QC H3A 0G1, Canada; Canadian Centre for Computational Genomics, McGill University, Montreal, QC H3A 0G1, Canada; Department of Human Genetics, McGill University, Montreal, QC H3A 0G1, Canada; Victor Phillip Dahdaleh Institute of Genomic Medicine at McGill University, Montreal, QC H3A 0G1, Canada; Canadian Centre for Computational Genomics, McGill University, Montreal, QC H3A 0G1, Canada; Department of Human Genetics, McGill University, Montreal, QC H3A 0G1, Canada; Victor Phillip Dahdaleh Institute of Genomic Medicine at McGill University, Montreal, QC H3A 0G1, Canada; Institute for the Advanced Study of Human Biology (WPI-ASHBi), Kyoto University, Kyoto 606-8303, Japan

## Abstract

**Motivation:**

Human epigenomic data has been generated by large consortia for thousands of cell types to be used as a reference map of normal and disease chromatin states. Since epigenetic data contains potentially identifiable information, similarly to genetic data, most raw files generated by these consortia are stored in controlled-access databases. It is important to protect identifiable information, but this should not hinder secure sharing of these valuable datasets.

**Results:**

Guided by the *Framework for responsible sharing of genomic and health-related data* from the Global Alliance for Genomics and Health (GA4GH), we have developed an approach and a tool to facilitate the exploration of epigenomics datasets’ aggregate results, while filtering out identifiable information. Specifically, the EpiVar Browser allows a user to navigate an epigenetic dataset from a cohort of individuals and enables direct exploration of genotype–chromatin phenotype relationships. Because individual genotypes and epigenetic signal tracks are not directly accessible, and rather aggregated in the portal output, no identifiable data is released, yet the interface allows for dynamic genotype—epigenome interrogation. This approach has the potential to accelerate analyses that would otherwise require a lengthy multi-step approval process and provides a generalizable strategy to facilitate responsible access to sensitive epigenomics data.

**Availability and implementation:**

Online portal: https://computationalgenomics.ca/tools/epivar; EpiVar Browser source code: https://github.com/c3g/epivar-browser; *bw-merge-window* tool source code: https://github.com/c3g/bw-merge-window.

## 1 Introduction

In recent years, multiple international consortia have been coordinating the mapping of the epigenetic landscape across human tissues and individuals. They include the Encyclopaedia of DNA Elements consortium (ENCODE) (ENCODE Project Consortium 2012), the NIH Roadmap (Bernstein *et al.* 2010) and the International Human Epigenome Consortium (IHEC) (Stunnenberg *et al.* 2016), which together have profiled the epigenetic features of over 3000 biosamples, in over 8000 epigenomic experiments. The hope is that these detailed epigenetic maps will lead to a better understanding of the human genome, including a better annotation of noncoding regulatory elements. The Genotype-Tissue Expression (GTEx) project goes a step further and is characterizing the effects of genetic variation on gene expression and chromatin (GTEx Consortium 2020). Through the identification of quantitative trait loci (QTLs) with either expression (eQTL) or chromatin (cQTL), the aim is to improve our understanding of the molecular mechanisms of genetic risk for complex traits and diseases. Notably, in line with open science principles and to facilitate further analyses and discoveries, the cited initiatives all provide access to various summary data on their portal. However, one of the main issues for researchers interested in these resources is that developing new visualizations or performing novel analyses on them can be challenging (Saulnier *et al.* 2019). That’s because the underlying raw data is hosted in registered or controlled-access databases, to protect potentially re-identifiable genetic data. In this context, we need better mechanisms to facilitate epigenomic data discovery and analysis, while addressing the ethical and privacy aspects associated with data sharing (Sharing Epigenomes Globally 2018).

Focusing mainly on genetic data, the Global Alliance for Genomics and Health (GA4GH) has been working on secure methods federating sensitive genomic data to facilitate data discovery. This includes the development of the Beacon v2 API specification (Rueda *et al.* 2022), defining a programming interface that enables third party tools and portals to discover and query the clinical and genomics content of project-specific databases. GA4GH has now also started to add API specifications for other -omics data, such as from RNA-seq experiments (Upchurch *et al.* 2023). Beyond supporting epigenomics data discovery, we wanted to enable data integration in a manner consistent with the GA4GH *Framework for responsible sharing of genomic and health-related data* (GA4GH 2019). Specifically, we wanted to facilitate genotype–chromatin phenotype relationships such as the ones identified by GTEx or IHEC (Aracena *et al.* 2024). Once a QTL is identified, we would facilitate the exploration of that locus and look at the underlying epigenomics data supporting an association. Currently, this can only be accomplished by a lengthy multistep process involving a data access-request, approval, download and an ad hoc analysis to group epigenomics tracks based on genotype. With the EpiVar Browser, we propose a seamless solution that presents summarized epigenomics data from a cohort faceted by genotype and allows direct visualization of merged signal tracks. Using the D-PATH policy assessment tool (Moreno *et al.* 2023), we ensured no identifiable information is released, meaning the data can be provided without a data access request. We still require users to authenticate with their IP address and agree to a terms of use document to ensure that data use conditions are acknowledged and respected.

## 2 Results

**Figure 1.**
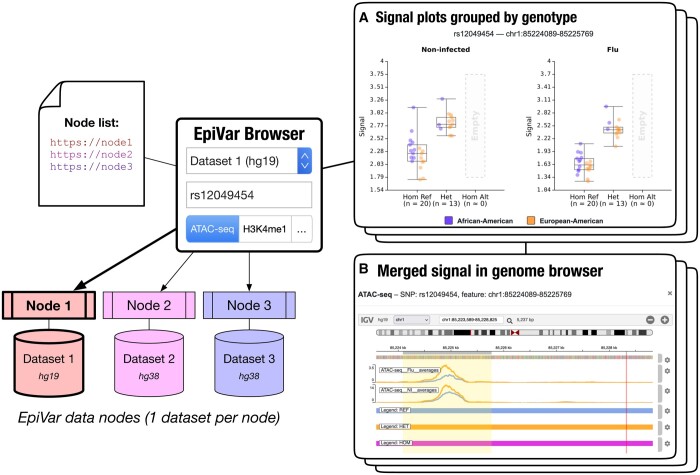
EpiVar Browser architecture. The EpiVar Browser interacts with multiple data nodes on secure web servers, each of which can generate summary data box plots on-the-fly using sample bigWig files for a selected assay and genotype information (A). In this example, the generated plots reveal correlations between the rs12049454 SNP genotype and chromatin accessibility in both noninfected and flu-infected cells. Genotype groups with insufficient sample count are masked. The merged signal for the feature, stratified by genotype, can be visualized in the UCSC Genome Browser or an integrated igv.js browser instance (B)

To demonstrate our proposed approach for allowing discovery of genetic-epigenetic relationships, we have implemented the EpiVar Browser, a portal that connects to a network of distributed nodes with access-controlled genetic and epigenetic datasets and enables researchers to explore the interaction between them. As a proof of concept, we set up two EpiVar Browser nodes using data with 510 epigenomics experiments from six different assays and 35 individuals obtained from a study exploring the response to influenza infection (Aracena *et al.* 2024) using the hg19 and hg38 (lifted over from hg19) reference genomes. This dataset contains over 5 million single nucleotide polymorphisms (SNPs) associated with ∼376 000 genomic features for a total of around 29 million associations at *P* < 0.05. Our web interface communicates with a set of data nodes, each hosting a single dataset—allowing for multiple datasets to be incorporated into the portal while keeping data processing and summarization as close to the data as possible. The data node server software supports datasets which use either the hg19 or hg38 reference genome. The interface allows users to provide a variant of interest, using its dbSNP accession ID (“rs” number) (Sherry *et al.* 2001), and suggests genomic features with the most significant associations (Supplementary Fig. S1). Alternatively, a user can provide the symbol for a gene of interest, after which they can choose a variant with relevant detected associations. The tool generates one boxplot per experimental condition (for these data, flu infection status) for each epigenetic assay available, stratified by genotype for that variant, with points coloured by population group (Fig. 1A). The list of conditions (such as flu infection status) and population groups (such as ethnicity) can be configured by data node administrators. Users can also visualize results in an integrated igv.js browser (Robinson *et al.* 2023) or in the UCSC Genome Browser (Kent *et al.* 2002), with tracks displaying the average of the experiment signal per genotype (Fig. 1B). These tracks are calculated upon request for a 200 kb genomic window centred on the feature of interest.

## 3 Implementation and compliance

The code for the EpiVar Browser is open source and released under the LGPL version 3.0 licence. The box plot charts are either created from precomputed point matrices or dynamically generated for the user-selected feature using the UCSC *bigWigSummary* tool (Kent *et al.* 2010). In either case, box plots are derived from locally stored signal and genotype data. Genotypes for all participants are stored in a compressed and Tabix-indexed (Li 2011) VCF file.

Signal tracks for all epigenetic experiments (one per assay/participant) are stored on an EpiVar data node in the bigWig format. Data processing is done at the hosting node, including the on-the-fly data processing and plot generation. Association significance for peak-SNP pairs must be computed ahead of time and imported into the portal. Precomputed signal values (to incorporate things such as batch correction) for feature-sample pairs can optionally be provided as well.

Browsing of tracks on the UCSC Genome Browser is made possible by producing a Track Hub with dynamically generated bigWig files for the genomic region of interest, per assay and per average signal for a genotype group. The original per-sample bigWig files were normalized so that each value represents the read count per base pair per 10 million reads. A Node.js application and a signal annotation file-processing service efficiently slices these bigWig files and merges them on demand, using our *bw-merge-window* utility. Genome browser tracks were created with the HOMER *makeUCSCfile* command and *bedGraphToBigWig* utility from UCSC (Kent *et al.* 2010). These tracks are created on the fly by averaging bigWig regions of samples sharing an experimental treatment and genotype. The track’s curve denotes the distribution of the average RPM values. A second track hub, which may be pre-configured by data node administrators, shows signals of various epigenetic marks coloured by condition.

During the implementation of the portal, we put particular attention into protecting the potentially privacy-compromising aspects of the data. Box plots are generated at the server-side, rather than presenting an API which may enumerate samples in a specific order, and the EpiVar Browser does not reveal individuals’ signal level/genotype pair with identifiers, so that the genotype data cannot be collected across multiple SNPs. If there are five or fewer samples in a particular genotype group, we do not reveal any genotype or signal data for the group, reporting it as “empty”. All of this is to prevent the re-identification of individuals using genetic information. Finally, we used the D-PATH privacy assessment tool to identify the applicable legal and ethical requirements regarding the sharing of epigenetic data and accompanying metadata (Moreno *et al.* 2023).

The raw data for genomic and epigenetic analyses stored in our EpiVar nodes are deposited at the European Genome-Phenome Archive (EGA). Researchers with an interest to get more information, such as participant-level granularity of the data, can apply for access to the relevant Data Access Committee.

## 4 Discussion

With the EpiVar Browser, potentially privacy-compromising data such as per-individual genotypes, usually stored under controlled access, are never directly accessible to the portal users; that information remains on each dataset nodes and computation is done at that location, generating on-demand the aggregate data to feed the portal. Variant information is made available only through genotype groups, and variant groups with five or fewer samples in them are not shown. In this way, we prevent users from discovering samples with known unique rare variants or aggregating genotypes or epigenetic data for a specific sample across multiple loci.

Our portal is implemented to allow integrating additional epigenomic datasets in the future. We have made available a Docker image with the EpiVar server code, allowing for others to run their own EpiVar node that could be connected to our portal. What we presented here also represents an exploration and visualization strategy that could be used more generally to accelerate access to sensitive epigenomics and other datasets.

## Supplementary Material

btae136_Supplementary_Data

## Data Availability

All the data used for the initial EpiVar Browser nodes are available under controlled access at the European Genome Phenome Archive (EGA) at https://ega-archive.org/datasets/EGAD00001008422 and https://ega-archive.org/datasets/EGAD00001008359, with the dataset IDs EGAD00001008422 and EGAD00001008359, respectively.
