# Supplementary material for: EpiVar Browser: advanced exploration of epigenomics data under controlled access
Source: Bioinformatics. 2024 Mar 6;40(3):btae136. doi: 10.1093/bioinformatics/btae136 (PMC10963074; doi:10.1093/bioinformatics/btae136)
Supplement: btae136_Supplementary_Data [file btae136_supplementary_data.pdf]

Supplementary material

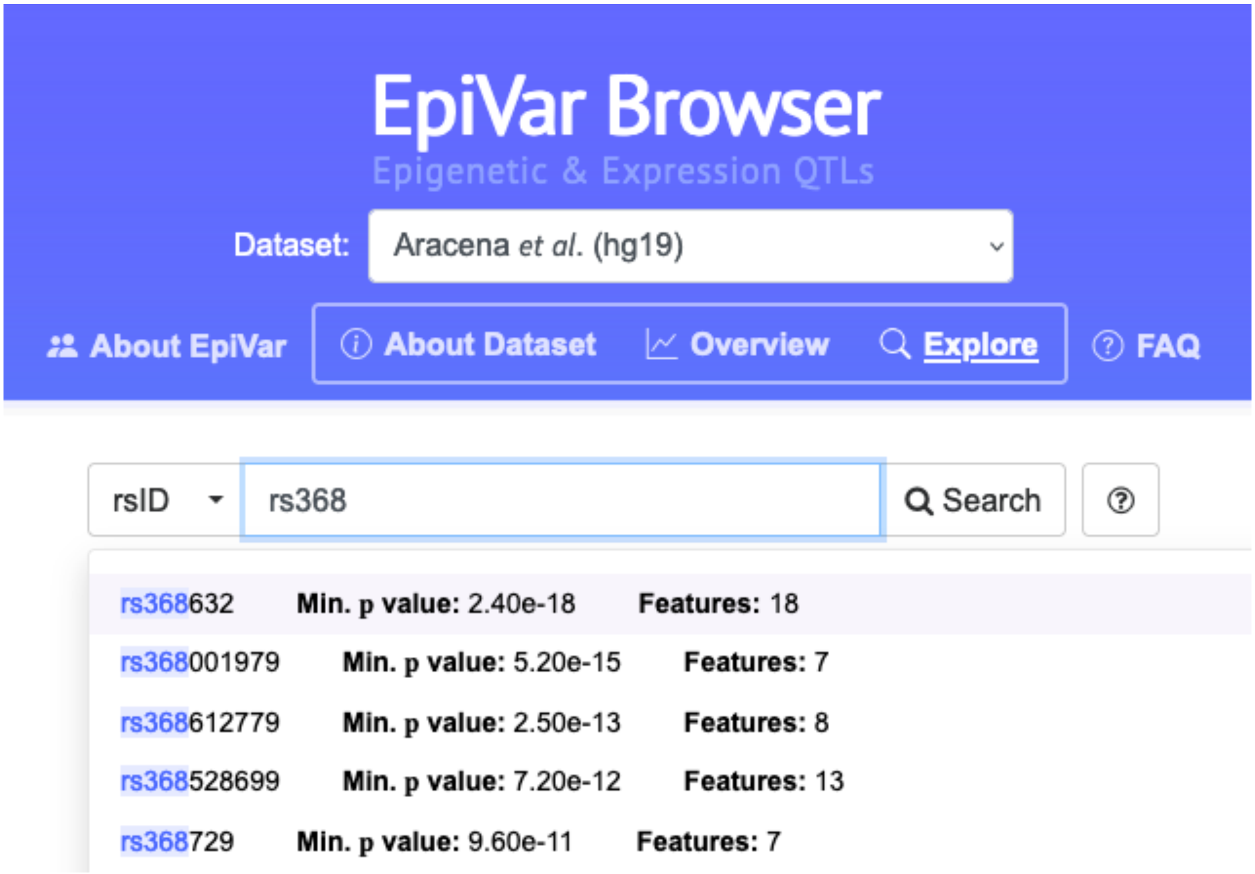

Figure S1: The EpiVar Browser allows SNP searching via rsID. Up to 50 matching results are shown; SNPs with the strongest available feature associations are shown first. Selecting a SNP will take a user to a page with the strongest peak, and the assay it came from, pre-selected.
